# Inflammatory hepatocellular adenoma in a patient with Turner’s syndrome: A case report

**DOI:** 10.1016/j.ijscr.2019.02.017

**Published:** 2019-02-15

**Authors:** Satoshi Nemoto, Shun-ichi Ariizumi, Yoshihito Kotera, Akiko Omori, Shingo Yamashita, Taka-aki Kato, Shota Aoyama, Hiroto Egawa, Masakazu Yamamoto

**Affiliations:** Department of Gastroenterology Surgery, Tokyo Women’s Medical University, Japan

**Keywords:** TS, Turner’s syndrome, HRT, hormone replacement therapy, HCA, hepatocellular adenoma, CT, computed tomography, MRI, magnetic resonance imaging, Gb-EOB-MRI, gadoxetic acid ethoxybenzyl magnetic resonance imaging, OCPs, oral contraceptive pills, CRP, C-reactive protein, LFABP, liver fatty acid-binding protein, GS, glutamine synthetase, IHCA, inflammatory hepatocellular adenoma, H-HCA, HNF1A-muted hepatocellular adenoma, RHV, right hepatic vein, b-HCA, β-catenin muted hepatocellular adenoma, UHCA, unclassified hepatocellular adenoma, TAE, trans-catheter arterial embolization, Case report, Turner’s syndrome, Hormone replacement therapy, Hepatocellular adenoma, Surgical resection

## Abstract

**Introduction:**

Hepatocellular adenoma (HCA) is a rare benign tumor and is related to the use of an oral contraceptive pill. Turner’s syndrome requires various hormone replacement therapies, including the pill which is used as a female hormone replacement therapy. Herein we report a case of Turner’s syndrome with HCA treated by liver segmentectomy.

**Presentation of case:**

A 36-year-old woman with Turner’s syndrome was treated with oral contraceptive pills as a female hormone replacement therapy for 20 years. She presented with fatigue and liver tumor. Liver tumors in the posterior lobe measuring 60 mm and 10 mm in diameter were detected on CT; hence, she was referred to our department. Both the tumors showed high intensity in the arterial phase, *iso*-intensity in the portal and late phases, and low intensity in the hepatobiliary phase on Gb-EOB-MRI. She was diagnosed with multiple HCAs and underwent segmentectomy Section 7. Pathologically, both the tumors were diagnosed as HCAs, and inflammatory markers were detected by immunohistochemistry. Thirteen months postoperatively, she was doing well and there was no evidence of recurrence of HCA without the pill.

**Discussion:**

There is only one report of HCA in patients with TS (Espat et al., 2000). We reported a case of multiple HCAs in a patient with TS underwent hepatectomy.

**Conclusion:**

With the use of the contraceptive pill as a long-term female hormone replacement therapy for Turner’s syndrome, careful attention is required for HCA.

## Highlights

• Contraceptive pill induced hepatocellular adenoma with Turner’s syndrome patient is extremely rare.
• However Turner’s syndrome patient requires a lifetime hormone replacement therapy.
• Proper diagnosis and treatment plan is necessary for hepatocellular adenoma.

## 1 Introduction

Hepatocellular adenoma (HCA) is a rare benign liver tumor, and the risk of using an oral contraceptive pill for HCA is well-known [2,3]· Turner’s syndrome (TS) is a genetic disorder with a completely or partly missing X-chromosome. It is usually associated with reduced height and gonadal dysgenesis; thus, patients with TS require a hormone replacement therapy (HRT), including a contraceptive pill, to support growth and maintain QOL [4,5]. To date, there is only one report of a patient with TS who developed HCA [1]. Here we report an extremely rare case of contraceptive pill-induced HCA with TS. The work in this case has been reported in line with the SCARE criteria [6].

## 2 Case presentation

The patient was a 36-year-old woman with Turner’s syndrome (TS) diagnosed at 9 years of age. She received an oral contraceptive pill as a hormone replacement therapy (HRT) from the age of 16 years. She was referred to our department in July 2017 after presenting at a local hospital with fatigue and liver tumors detected on computed tomography (CT).

**Fig. 1:**
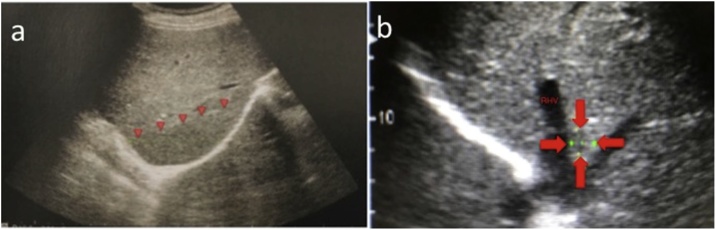
Abdominal ultrasonography showing (a) a hypoechoic lesion measuring 61 mm in diameter located in the posterior section and (b) a hypoechoic lesion measuring 10 mm in diameter at the root of the right hepatic vein.

Her physical examination and blood tests showed no remarkable findings. Abdominal ultrasonography showed a low echoic tumor, which was 60 mm in diameter, in the posterior section of the liver and an isoechoic tumor, which was 6 mm in diameter, at the root of the right hepatic vein (RHV; Fig. 1).

**Fig. 2:**
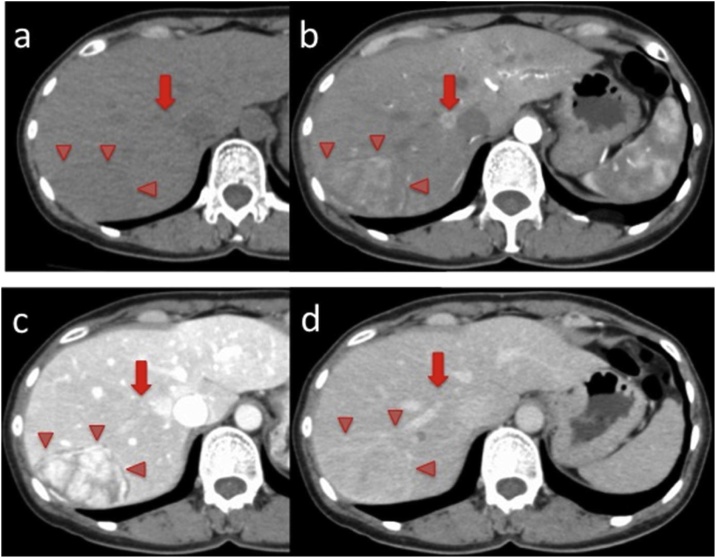
Plain and enhanced CT findings. A 61 mm tumor in the posterior section was observed as (a) a low density area in the plain phase, (b) a slightly high density area in the artery phase, (c) a strong high density area in the portal phase, and (d) an isodensity area in venous phase. A 10 mm tumor at the root of right hepatic vein was observed as (a) a low density area in the plain phase, (b) a high density area in the artery phase, (c) a high density area in the portal phase, and (d) an isodensity area in the venous phase.

CT showed a 60-mm tumor in the posterior section of the liver. This tumor showed high density in the arterial phase and isodensity in the portal and late phases. Another small 10-mm tumor at the root of RHV also showed high density in the arterial phase and isodensity in the portal and late phases (Fig. 2).

**Fig. 3:**
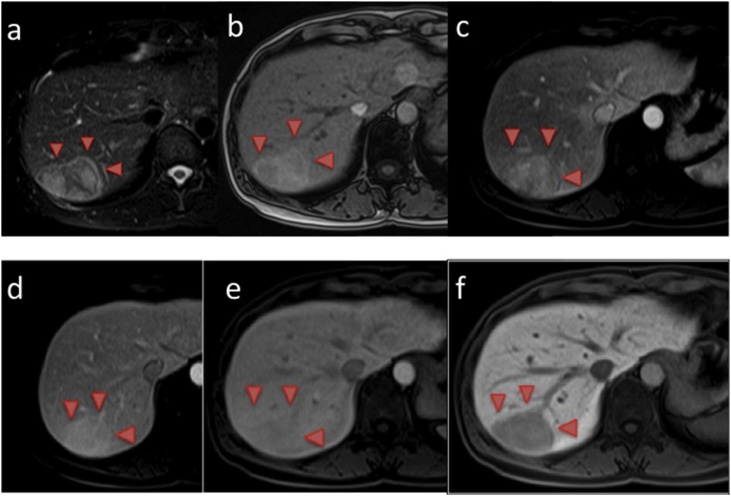
Gb-EOB-MRI findings. The 61 mm tumor in the posterior section showed high intensity on (a) T2-weighted and (b) pre dynamic images. A high intensity area in the arterial phase was also observed that washed out in 60 s (c) to 180 s (e). In the hepatobiliary phase, a borderline, clear, low intensity lesion is seen (f). The small 11 mm tumor could not be seen by Gb-EOB-MRI.

Gadoxetic acid ethoxybenzyl magnetic resonance imaging (Gb-EOB-MRI) of the large tumor showed high intensity on T2-weighted images and in the arterial and portal phases, and low intensity in the late and hepatobiliary phases, while the small tumor showed low intensity in the hepatobiliary phase (Fig. 3).

Based on the diagnosis of multiple HCAs or hepatocellular carcinomas (HCCs), segmentectomy of No 7 of the liver was performed. The operation time was 178 min, and blood loss was 681 mL.

**Fig. 4:**
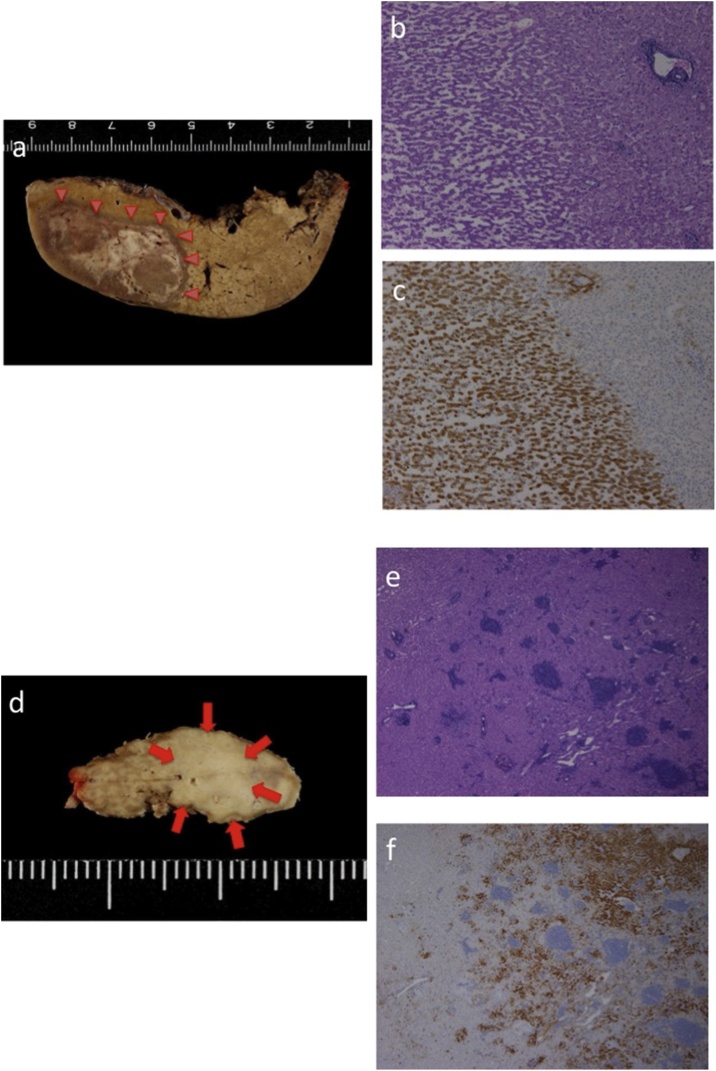
Pathological and immunohistochemistry findings. Regarding the larger tumor, (a) macroscopically, it measured 61 mm and was white in color, (b) microscopically, it had normal hepatocytes with mild sinusoidal dilation, and (c) the hepatocytes were positive for CRP by immunohistochemistry. The smaller tumor measured 11 mm in diameter with (d) a white area and an unclear margin, (e) normal hepatocytes and lymphocytes, and (f) CRP-positive hepatocytes.

Macroscopic findings showed a whitish and brownish tumor, which was 61 mm in diameter and without capsula and another small, whitish, 11-mm tumor without capsula. Pathological findings of the larger tumor showed hepatocytes without atypia, with sinusoid dilatation and a single vessel seen within the tumor. This tumor was diagnosed as HCA. Immunohistochemistry findings of the larger tumor showed that the hepatocytes were positive for C-reactive protein (CRP) and liver fatty acid-binding protein (LFABP) and negative for β-catenin, glutamine synthetase (GS), and glypican-3 (Fig. 4). The small tumor showed same pathological and immunohistochemistry findings; therefore, both the tumors were diagnosed as inflammatory HCA (IHCA).

The patient was discharged on postoperative day 14. At the 13-month postoperative follow-up, she was doing well and there was no evidence of recurrence of HCA without the pill.

## 3 Discussion

TS requires various HRTs, with the contraceptive pill often administered [4,5], which is associated with the very rare benign liver tumor HCA [2,3]. The incidence rates of HCA with the long-term use of oral contraceptive pills are approximately 3–4 per 100,000 individuals [7]. There is only one report of HCA in patients with TS [1]; we reported a case of multiple HCAs in a patient with TS.

In 2010, the World Health Organization classified HCA into four subtypes based on immunohistochemistry findings: IHCA, HNF1A-mutated HCA (H-HCA), β-catenin-mutated HCA (b-HCA), and unclassified HCA (UHCA) [8,9]. The majority of pill-induced HCAs are IHCAs [10]. This is consistent with the diagnosis of IHCA in our present case.

Patients with TS experience various complications [4], commonly including abnormal liver function [11,12], congenital portal vein deficiency, focal nodular hyperplasia (FNH), nodular regenerative hyperplasia (NRH), and HCC [[13], [14], [15]]. However, imaging and pathological findings for FNH, NRH, HCA, and HCC are similar, which can result in difficulties in the differential diagnosis. Therefore, the risk of not only benign liver tumors (FNH, NRH, and HCA) but also HCC should be considered in TS.

Indication of treatment for HCA has been reported [[16], [17], [18]]. When the tumor size is <5 cm in diameter, follow-up is recommended after discontinuing the medication, including the pill. When the tumor size is ≥5 cm, hepatectomy is recommended because HCAs pose a risk of rupture and can transform into HCC [16,18,19]. In the present case, hepatectomy was performed because there were multiple tumors, and the largest tumor measured 6 cm. The contraceptive pill as a female HRT was discontinued postoperatively to prevent the recurrence of HCA.

## 4 Conclusion

We experienced a case of multiple inflammatory HCAs in TS that were related to the long-term use of a contraceptive pill. Therefore, careful attention is required for HCA in patients with TS taking contraceptive pills as a long-term female HRT.

## Conflicts of interest

No conflict of interest.

## Sources of funding

No funding.

## Ethical approval

This case report is exempt from ethical approval by our institution.

## Consent

Written informed consent was obtained from the patient for publication of this case report and accompanying images. A copy of the written consent is available for review by the Editor-in -Chief of this journal on request.

## Author contribution

Satoshi Nemoto: treated and helped operation, collected the data, and drafted the manuscript.

Shun-ichi Ariizumi: conducted the examinations on this patient as well as treated and operated, drafted the manuscript, pathological examination and contributed to the diagnosis of this patient.

Yoshihito Kotera, Akiko Omori, Taka-aki Kato and Shota Aoyama: helped treat this patient.

Hiroto Egawa: conducted the manuscript.

Masakazu Yamamoto: conducted the manuscript and operate the patient.

All authors read and approved the final manuscript.

## Registration of research studies

No registration of research studies.

## Guarantor

On behalf of all author, Yamamoto Masakazu M.D. is guarantor for this paper.

## Provenance and peer review

Not commissioned, externally peer-reviewed.
